# Effect of Water Activity on Conidia Germination in *Aspergillus flavus*

**DOI:** 10.3390/microorganisms10091744

**Published:** 2022-08-29

**Authors:** Sifan Jia, Chong Li, Kuntan Wu, Desheng Qi, Shuai Wang

**Affiliations:** Department of Animal Nutrition and Feed Science, College of Animal Science and Technology, Huazhong Agricultural University, Wuhan 430070, China

**Keywords:** water activity, *Aspergillus flavus*, conidia germination, transcriptomics, proteomics

## Abstract

In this study, we explored the mechanism underlying *Aspergillus flavus* conidia germination inhibited by decreased water activity. The impact of low water activity was analyzed at 4 h, 8 h and 12 h. Additionally, we demonstrated that low water activity affected cell shape and decreased cell sizes. Transcriptomics found numerous differentially expressed genes (DEGs) during the first 12 h of germination, with 654 DEGs observed among 4 h, 8 h and 12 h. In particular, more DEGs were detected at 8 h of germinating. Therefore, proteomics was performed at 8 h, and 209 differentially expressed proteins (DEPs) were speculated, with 94 up-regulated and 115 down-regulated. Combined analysis of KEGG of transcriptomics and proteomics demonstrated that the dominant pathways were nutrient metabolism and translation. We also found several DEGs and DEPs in the Mitogen Activated Protein Kinase (MAPK) pathway. Therefore, we concluded that low water activity inhibited conidia germination, causing unregular morphology. In addition, low water activity influenced expression of *creA*, *TreB* in carbohydrate metabolism, *Clr4*, *RmtA* in amino acid metabolism and *RPL37, RPL3* in translation in *Aspergillus flavus*.

## 1. Introduction

*Aspergillus flavus* is a pathogen that widely pollutes food and feed crops, including corn, peanuts, cotton and maize [1]. This fungus produces mycotoxins, such as aflatoxin B_1_, that causes harmful disease to animals, plants and insects [2]. Therefore, seeds infected by *A. flavus* could result in great damage to agricultural and economic losses by diminishing values and lower price of the crops [3].

Previous research [4,5] demonstrated that water activity (a_w_) is an important environmental factor for fungi. A_w_ is an important index that describes the state of water [6]; it is a measurement of freely available water required for microorganisms to grow, and it relates to pure water, which contains an a_w_ of 100% relative moisture [7]. Accumulating evidence [8,9] has indicated that a_w_ could affect the germination, growth and toxigenicity of *A. flavus*. In addition, many studies [4,10,11] have confirmed that a_w_ is important for germination of conidia of *Aspergilli*. Moreover, a germination model of fungal conidia was established, which [12] concluded that conidia germination was affected by temperature, percentage of germinated conidia and fungal strains to a great extent. The study created a model that could effectively be used as a quality management system for fungus control. Research performed on *A. fresenii* and *A. sulphureus* [4] revealed the optimum a_w_ and temperature for both fungi to grow, but did not focus on conidia germination, limiting the category of a_w_ and temperature.

Transcriptomics was performed in *A. flavus* to reveal the relationship between toxigenicity and a_w_ [9], with data corroborating that a_w_ had an impact on aflatoxin biosynthesis. Research on *Aspergillus niger* [13] found that low a_w_ causes damage to hyphae. A recent study on the interaction of temperature and a_w_ on *A. flavus* [14] reported that the optimum conditions for the fungi to grow were at 0.92–0.96 a_w_ and 28–37 °C. Moreover, research [15] has demonstrated the effect of a_w_ and temperature on the growth and aflatoxin production of *Aspergillus parasiticus* and *A. flavus*.

Transcriptomics of *A. niger* conidia was analyzed during the first 8 h of germination and 4780 expressed genes were observed [16]. The study concluded that conidia change mainly in protein synthesis, cell cycle, DNA processing, respiration and metabolism. A study of *A. flavus* [17] demonstrated that the MAPK pathway is important in the regulation of development, secondary metabolism and pathogenicity. Moreover, proteomics was used to evaluate *A. flavus* in response to a_w_ [18], revealing that at two different a_w_, 837 differentially expressed proteins were identified. Another proteomic study on *A. flavus* [19] found 417 identified proteins in germinating conidia, and functional characterization of proteins were mostly to cell wall synthesis and degradation, metabolisms, protein synthesis and degradation. A study of proteomics of *A. fumigatus* conidia-containing phagolysosomes [20] identified 637 host and 22 proteins that were differentially abundant in phagolysosome and found several key proteins in some processes, including signaling pathways and so on.

Other fungal species have been studied extensively in regard to conidia germination, such as *A. nidulans* [21], *A. niger* [16], *A. fumigatus* [22] and *yeast* [23]. Little attention, however, has been devoted to the conidia germination of *A. flavus*. Our research detects the blind spots of previous studies of *A. flavus*, and we investigated the effect of a_w_ on conidia germination. Finding methods to reduce toxin produced by *A. flavus* is an efficient way to solve the contamination by fungal toxin. Thus, when the mechanism of conidia germination is cleared, the problem could be solved from the origin state. As a result, we investigated the relationship between a_w_ and conidia germination by transcriptomics and proteomics, attempting to figure out the molecular mechanism by which a_w_ influences the conidia germination of *A. flavus*.

## 2. Materials and Methods

### 2.1. Fungal Strain and Growth Conditions

The *Aspergillus flavus* NRRL 3357, kindly provided by Zhumei He (Sun Yat-sen University, Guangzhou, China) was used in this study. Conidia were cultured on fresh potato dextrose agar medium at 30 °C for seven days, then collected and suspended in sterile water containing 0.05% Tween-80. The conidia suspension was filtered through sterile four layers lens paper and kept on ice until further processing on the same day. Conidia were counted using a hemocytometer and diluted to 10^6^ per mL. The corresponding a_w_ of the medium was adjusted to 0.98 a_w_ and 0.90 a_w_ as in previous studies (with glycerol 13.1 mL, 0.98; 31.3 mL, 0.90) [24]. Water activity was verified using a water activity meter (LabMaster−a_w_, Novasina, St. Gallen, CH).

After incubating at different a_w_ levels, this strain was used for different purposes. For conidia germination, 10 µL conidia’ suspension was added to YES agar medium (150 g source, 20 g yeast extract and 15 g agar per liter) with 6 cm Petri plates and cultured at 30 °C for 4, 8 or 12 h, then a microscope was utilized to observe different germination states of the conidia. For RNA extraction, 20 mL conidia’ suspension was added to 200 mL YES medium, shaking at 150 rpm at 30 °C for 4, 8 or 12 h, after which the liquid medium was centrifugated at 3000 rpm and the conidia then collected.

To determine the rate of germination, a total of at least 200 conidia per sample were examined. Conidia were considered as germinated if a germ tube of at least 2 μm was visible.

### 2.2. Microscopy

The specific process is displayed in the Appendix A. Briefly, the conidia were harvested after incubating for 4 h, 8 h and 12 h. An optimal microscope was utilized for direct observation and images of the conidia. With a scanning electron microscope (SEM), the conidia were washed and immediately fixed with 2.5% glutaraldehyde for two hours, then transferred at 4 °C for preservation and transportation. The conidia were then washed with several reagents (details in Appendix A) and dried with a Critical Point Dryer, then attached to metallic stubs with gold for 30 s to observe and take images.

### 2.3. Flow Cytometry of Conidia

Flow cytometry was used to measure the size of the conidia over the first few hours of germination. Liquid medium was inoculated with conidia at a concentration of 10^6^ per mL, shaking at 150 rpm at 30 °C. The conidia were harvested by centrifuge (3000 rpm, five minutes) after inoculating for 4 h and 8 h. The supernatant was removed and conidia were washed in 1 mL Tween 80 (0.01% *v*/*v*). The samples were then analyzed using flow cytometry (Beckman-CytoFLEX Coulter, Brea, CA, USA) to determine the forward scatter (FSC) parameter for each sample to measure the size of the conidia.

### 2.4. RNA-Seq Analysis

The specific process of RNA-Seq analysis is detailed in the Appendix A. In brief, the total RNA of 4 h, 8 h and 12 h conidia were extracted using TRIzol^®^ Reagent (Plant RNA Purification Reagent for plant tissue; Invitrogen, Waltham, MA, USA), according to the manufacturer’s instructions. RNA-seq transcriptome library was prepared following TruSeqTM RNA sample preparation Kit from Illumina (San Diego, CA, USA) using 1 μg of total RNA. The raw paired end reads were trimmed and quality controlled by SeqPrep (https://github.com-/jstjohn/SeqPrep) (accessed on 2 July 2020) and Sickle (https://github.com/n-ajoshi/sickle) (accessed on 2 July 2020) with default parameters. Then clean reads were separately aligned to reference genome with orientation mode using HISAT2 software (version 2.1.0) (http://ccb.jh-u.edu/software-/hisat2/index.shtmL) (accessed on 8 July 2020) [25]. The mapped reads of each sample were assembled by StringTie (https://ccb.jhu.edu/software/stringtie-/index.shtmL?t=example) (accessed on 10 July 2020) in a reference-based approach [26]. To identify DEGs (differential expression genes), DESeq analysis was used. Transcriptomics was considered to have significant differential genes if fold change (>2) and *p*-value < 0.05. In addition, functional-enrichment analysis including GO [27] and KEGG [28] was performed to identify which DEGs were significantly enriched in GO terms and metabolic pathways by Fisher’s exact test at Bonferroni-corrected *p*-value ≤ 0.05 compared with the whole-transcriptome background. GO (version 2020.0628) functional enrichment and KEGG (version 2020.07) pathway analysis were carried out by Goatools (version 0.6.5) (https://github.com/tanghaibao/Goatools) (accessed on 25 July 2020) and KOBAS (version 2.1.1) (http://kobas.cbi.pk-u.edu.cn/home.do) (accessed on 25 July 2020) [29].

### 2.5. Real-Time Quantitative PCR

The quality and amount of total RNA was measured by NanoDrop 2000 spectrophotometer (Thermo Fisher Scientific, Walthamm, MA, USA). The first-strand cDNA was synthesized from the extracted RNA using a PrimeScript^™^ RT reagent Kit (Takara, Kusatsu City, Japan). Real-time quantitative PCR was conducted on a Bio-Rad CFX384 Real-Time PCR System with TB Green^®^ Premix Ex Taq™ II (Tli RNaseH Plus) (Takara). The relative amounts of mRNAs were normalized with the housekeeping gene β-actin and were analyzed by the 2^−ΔΔCt^ method [30]. All primer information were shown in Table S1.

### 2.6. Tandem Mass Tag (TMT)-Labelling Analysis

The specific process of the TMT-labeling analysis is outlined in the Appendix A. In brief, a total protein of eight-hour conidia were extracted from *A. flavus* by using a urea lysis buffer (7 M urea, 2 M thiourea, and 1% SDS) with a protease inhibitor. Protein concentrations were detected with a BCA Protein Assay Kit (Thermo, Grand Island, NY, USA). Following reduction, cysteine alkylation, and digestion, the samples were labelled with TMT reagent (Thermo Fisher, Art.No.90111, Walthamm, MA, USA) according to the manufacturer’s instructions. After being desalted with a C18 solid-phase extraction, peptides were used for Nano Liquid Chromatography–Mass Spectrometry/Mass Spectrometry (LC–MS/MS) analysis [31]. Labeled peptides were analyzed by online nano flow liquid chromatography tandem mass spectrometry performed on a 9RKFSG2_NCS-3500R system (Thermo, Bellefonte, PA, USA) connected to a Q Exactive Plus quadrupole orbitrap mass spectrometer (Thermo, Bellefonte, PA, USA) through a nano electrospray ion source. The RAW data files were analyzed using Proteome Discoverer (Thermo Scientific, Version 2.2) against *Aspergillus flavus* database (Aspergillus_flavus.JCVI-afl1-v2.0.pep.unique.fa.MJ20200708085.fasta). A total of 5460 proteins expressed were identified as belonging to the proteome of *A. flavus* in this study. The thresholds of fold change (>1.5 or <0.67) and *p*-value < 0.05 at Student’s *t*-test were used to identify differentially expressed proteins (DEPs). Then we found 115 up-regulated and 94 down-regulated proteins in 0.98 a_w_ group compared with 0.90 a_w_ group. In addition, functional-enrichment analysis of GO was performed to identify which DEPs were significantly enriched in GO terms by Fisher’s exact test at Bonferroni-corrected *p*-value ≤ 0.05 compared with the whole-proteomic background.

### 2.7. Statistical Analysis

One-way analysis of variance (ANOVA) was used to test the effects of a_w_. The analyses were conducted using the SPSS Statistics 21.0 package (SPSS Inc., IBM, New York, NY, USA). The figures were generated using GraphPad Prism 6 (Graph Pad Software Inc., San Diego, CA, USA), and processed using Adobe Illustrator CC 2019 (Adobe Systems Inc., San Jose, CA, USA). Annotation of all identified proteins was performed with Gene Ontology (GO; http://www.blast2go.com/b2ghome; http://geneontology.org/) (accessed on 3 October 2020) and KEGG pathway (http://www.genome.jp/kegg/) (accessed on 3 October 2020). Flow cytometry was analyzed using FlowJo_V10 (version 10.8, Becton, Dickinson and Company, 2021).

## 3. Results

### 3.1. Conidia Germination

**Figure 1:**
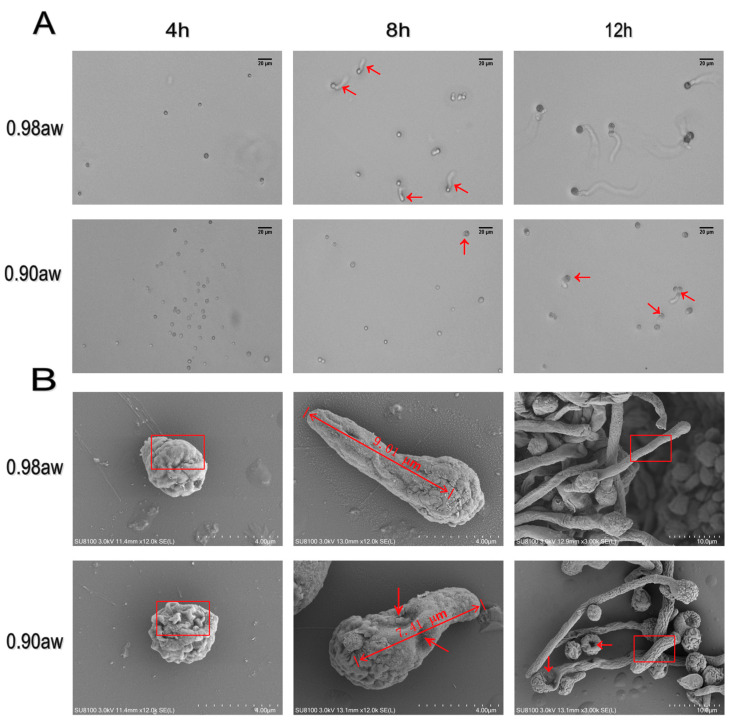
Germination of *A. flavus* conidia as observed by microscopy. The states of 4 h, 8 h and 12 h were observed by different kinds of microscopy. All conidia were observed under two different a_w_, 0.98 and 0.90. Germination of *A. flavus* conidia under two different a_w_ in optical microscope (**A**), bar represents 20 µm; in SEM (scanning electron microscope) (**B**), bar represents 4 µm in 4 h and 8 h, 10 µm in 12 h.

In this study, *A. flavus* was grown at 30 °C, enabling the separation of different stages of germination in time. Based on previous studies [32] and our observation, we chose 4 h, 8 h and 12 h to represent states of swelling, germ tube formation and mitosis. The germination rate of conidia was observed and listed in Figure S1. To clearly investigate the morphology of conidia germination, two types of microscopy (light microscope and SEM) and three different germination stages were observed. Conidia germination is shown in Figure 1A; no morphological changes were observed at 4 h of germination, and conidia were in the stage of swelling. While differences occurred at 8 h, that isotropic growth was observed in the 0.98 a_w_ group, several conidia germinated and germ tubes were formed (conidia with red arrows in 8 h), while most of the 0.90 a_w_ group was still in the stage of swelling. At 12 h, long mycelium was shown in the 0.98 a_w_ group, while still no more than half of the conidia germinated in the 0.90 a_w_ group (conidia with red arrows in 12 h). The SEM (Figure 1B) shows that in the 0.98 a_w_ group, the conidia were relatively smooth, and the shape of the cells were relatively regular. In comparison, in the 0.90 a_w_ group, it was observed that low water activity caused many folds in the cell surface. The differences were clearly shown between two red boxes. In addition, cells possessed with relatively rough surface and significant sinking (marked by red arrows) could be visualized in some conidia at 8 h and 12 h. Moreover, at 8 h, the germ tube was longer in the 0.98 a_w_ group (measured with segment in 8 h).

### 3.2. Flow Cytometry of Conidia

**Figure 2:**
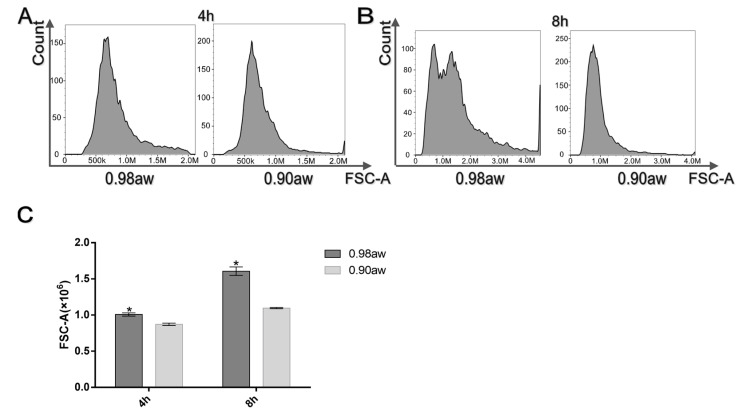
Flow cytometry data. The differences in size of conidia germination under two a_w_ and two states. The *x*-axis indicates forward scatter (FCS), the *y*-axis indicates counts of profiles of 10,000 conidia at 4 h (**A**) and 8 h (**B**). (**C**) Average size of 10,000 conidia measured as the FSC parameter. The means and standard errors of duplicate samples have been plotted (*n* = 3). Mean values with * were significantly different (*p* < 0.05).

In order to measure the differences in size of the conidia due to low a_w_, conidia were prepared and analyzed by flow cytometer over a period of 8 h. The counts of conidia demonstrated that compared to the 0.98 a_w_ group, more conidia were smaller at 4 h and 8 h in the 0.90 a_w_ group (Figure 2A,B). To illustrate this, we produced a graph based on the value of FSCs of the samples and found a significant decrease in the size of the conidia under low a_w_ in both groups (Figure 2C).

### 3.3. Summary of DEGs

**Figure 3:**
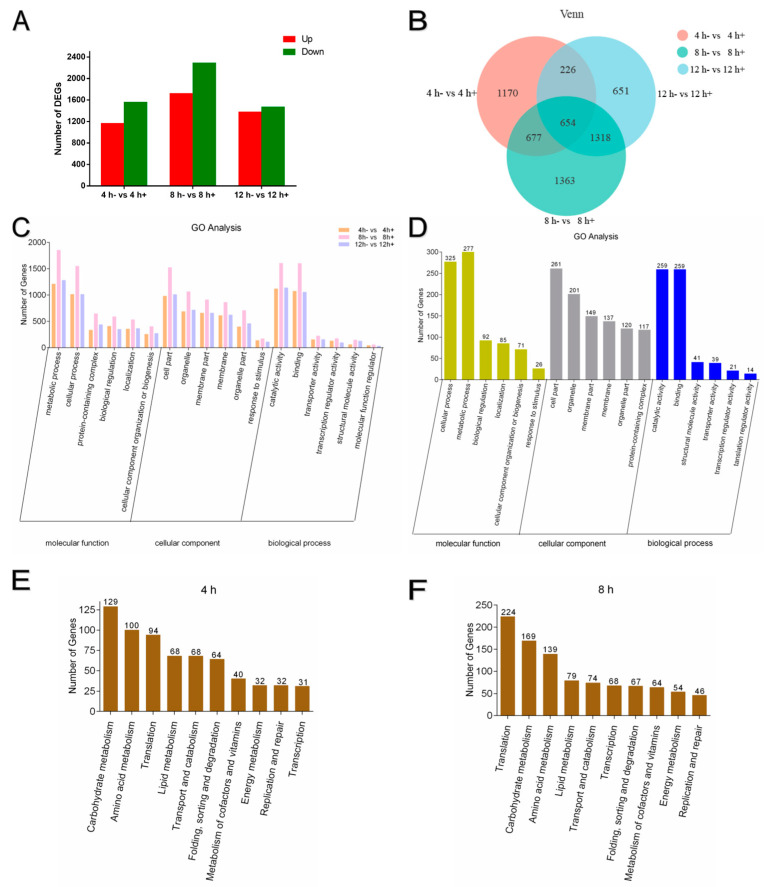

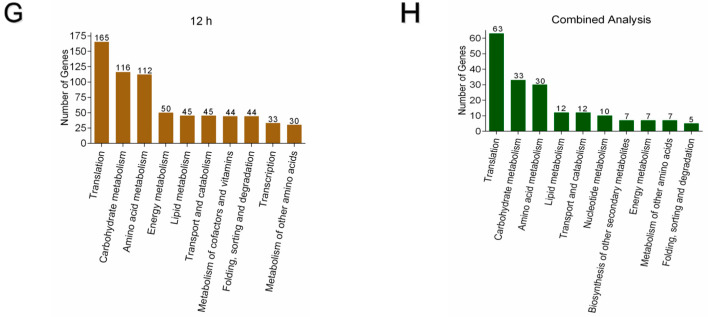
Summary of regulated genes. − (0.98 a_w_) and + (0.90 a_w_) represented two different a_w_; the value of gene expression is significantly (*p* < 0.05) differentially expressed (>two-fold) between the two groups. (**A**) Number of different expressed genes (DEGs) in each group; the red plot indicates up-regulated gene number and the green plot indicates down-regulated number. (**B**) Venn of DEGs in each group. (**C**) Top six of Gene Ontology (GO) categorization of three independent groups. The *x*-axis indicates the subcategories, and the *y*-axis indicates the number of genes in the same category. (**D**) Top six of GO categorization of combined analysis of three different groups. The red columns represent molecular function, green columns represent cellular component, blue columns represent biological process. Top 10 of KEGG pathways in 4 h (**E**), 8 h (**F**) and 12 h (**G**) group. The *x*-axis indicates the pathways, and the *y*-axis indicates the number of genes. (**H**) Top 10 of KEGG pathways of combined analysis of three groups.

Transcriptomic data show the regulated genes, together with Gene Ontology (GO) and KEGG analysis in Figure 3. The principal component analysis (PCA) indicated that transcriptomic data in the same groups had good repeatability (Figure S2), and significant differences were shown between each group (Figure 3A and Figure S2). Numerous length distribution of assembled unigenes was identified in transcriptomics, ranging from 0–1800 bp (Figure S3). A summary of DEGs (Figure 3A) revealed that regulated genes differed most at 8 h (n = 4012), with 1722 up-regulated and 2290 down-regulated. While the number of regulated genes was similar at 4 h and 12 h, with 1167 up-regulated and 1560 down-regulated at 4 h (n = 2727), 1378 up-regulated and 1471 down-regulated at 12 h (n = 2849). According to the above results, we assumed that the influence of *A. flavus* conidia germination by low a_w_ may be greater at 8 h compared to 4 h and 12 h. The Venn diagram (Figure 3B) showed 654 DEGs in common among these three groups. GO analysis was then applied to classify the functions of DEGs in each group. The results were summarized in the three main GO categories: molecular functions, cellular components and biological process. Several significantly enriched terms in the three categories were identified (*p* < 0.05), and the top six of each category (Figure 3C) for three different groups were listed. It was revealed that the dominant terms in the comparisons of the 0.98 a_w_ group to 0.90 a_w_ group were cellular progress, metabolic progress, cell part, binding and catalytic activity. In addition, data revealed that at 8 h, DEGs were much more than 4 h and 12 h, indicating more changes may have occurred at 8 h. Moreover, another GO analysis was performed regarding the common 654 DEGs to determine their function in conidia germination (Figure 3D). Regardless, we listed the top six of each category. It was revealed that the dominant terms in the comparisons of the 0.98 a_w_ groups to 0.90 a_w_ groups were cellular progress, metabolic progress, cell part, organelle, catalytic activity and binding, with results mostly consistent with the previous analysis. Moreover, KEGG analysis was followed. The top 10 pathways of DEGs at 4 h (Figure 3E), 8 h (Figure 3F) and 12 h (Figure 3G) were listed, as well as the common DEGs of the three groups (Figure 3H). The histograms showed consistency in the top five pathways for all the groups, which were translation, carbohydrate metabolism, amino acid metabolism, lipid metabolism, and transport and catabolism. In addition, the results showed that the dominant pathways for all the groups were translation, carbohydrate metabolism and amino acid metabolism. Among the translation pathways, DEGs were most enriched in ribosome and ribosome biogenesis in eukaryote pathways, with 18 genes and 35 genes significantly changed by low a_w_. For carbohydrate metabolism, 33 genes were regulated in carbon metabolism and 9 genes were regulated in starch and sucrose metabolism. In amino acid metabolism, 7 genes were regulated in arginine and proline metabolism, 2 genes were regulated in arginine biosynthesis pathway, and 5 genes were regulated in tryptophan metabolism.

### 3.4. Real-Time Quantitative PCR

**Figure 4:**
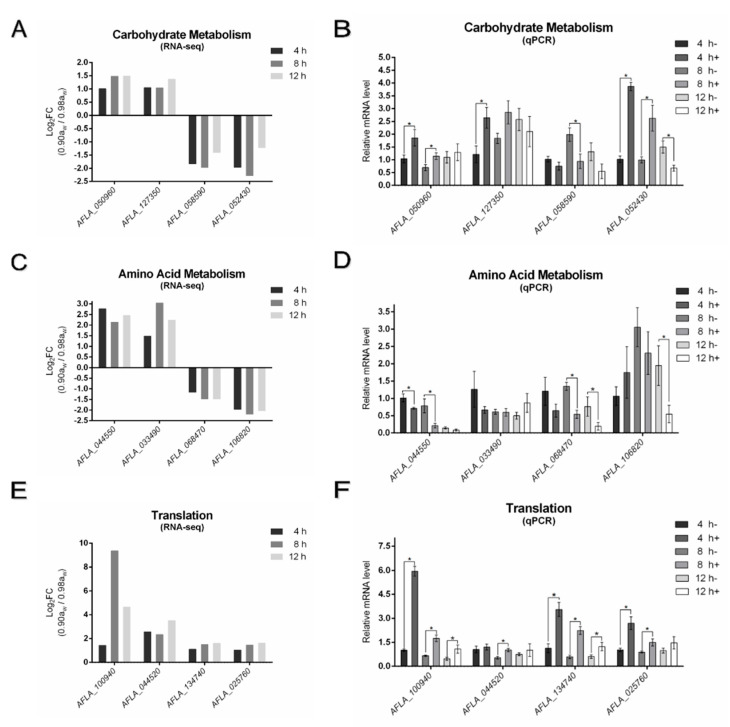
Fold change (FC) and real-time quantitative PCR of high regulated genes in three dominant pathways. − (0.98 a_w_) and + (0.90 a_w_) represented two different a_w_. Fold change of regulated genes in carbohydrate metabolism (**A**), amino acid metabolism (**C**) and translation (**E**). Real-time quantitative PCR of the four genes in carbohydrate metabolism (**B**), amino acid metabolism (**D**) and translation (**F**). The means and standard errors of duplicate samples have been plotted (*n* = 6). Mean values with * were significantly different (*p* < 0.05).

Based on our transcriptomics data, we found that the dominant pathways were carbohydrate metabolism, amino acid metabolism and translation. Therefore, real-time quantitative PCR was performed to validate the data from the transcriptome (Figure 4). We verified the top two up-regulated and down-regulated genes in each pathway, and since all genes were up-regulated in the translation pathways, we verified the top four up-regulated genes in the translation pathway. In addition, fold changes (FC) of the matched genes are listed. The results were highly consistent with the transcriptomics data and none of the RNA-seq comparisons (log_2_FC) were significantly different (*p* < 0.05) from the qPCR results, except only one gene (*AFLA_044550*) in the amino acid metabolism pathway was down-regulated in real-time quantitative PCR, while up-regulated in transcriptomics.

### 3.5. Summary of DEPs

**Figure 5:**
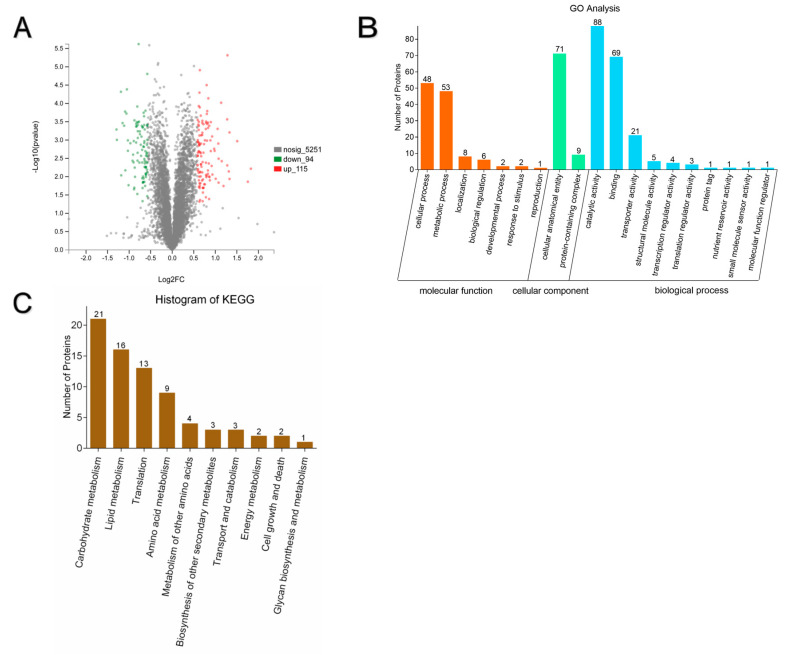
Summary of regulated proteins. The value of protein expression is significantly (*p* < 0.05) differentially expressed (>1.5-fold) between two groups. (**A**) The volcano plot of regulated proteins. Red dots represent up-regulated, green dots represent down-regulated and grey dots represent no significance (compared to the 0.98aw group). The *x*-axis indicates fold change (log_2_FC), and the *y*-axis indicates *p*-value (log_10_ *p*). (**B**) GO categorization of different expressed proteins of two a_w_ groups (**C**) Top 10 of KEGG pathways of different expressed proteins. The *x*-axis indicates the pathways, and the *y*-axis indicates the number of proteins.

The transcriptomics results revealed that compared to 4 h and 12 h, more DEGs were shown at 8 h, with approximately 1.5-fold, which indicated that more biological processes may be influenced by low a_w_ at 8 h. Therefore, proteomics was used at 8 h to further profile the mechanism of conidia germination influenced by low a_w_. The PCA analysis indicated that the proteomics data in the same groups had good repeatability (Figure S4), and significant differences were shown between each group (Figure 5A and Figure S4). Using TMT-labeled peptides, numerous different peptide length distributions were identified (Figure S5), as well as a total of 5592 proteins, and the coverage distribution of the identified proteins is shown (Figure S6). In total, 209 proteins were significantly (*p* < 0.05) changed (>1.5-fold) between the two groups. Among these 209 proteins, 115 were significantly up-regulated in the 0.90 a_w_ group, and 94 were significantly down-regulated (Figure 5A). Furthermore, GO analysis was applied to classify the functions of the proteins (Figure 5B). Several significantly enriched terms (*p* < 0.05) in the three categories were identified in comparisons of the 0.98 a_w_ groups and 0.90 a_w_ groups. It was revealed that the dominant terms were cellular process, metabolic process, cellular anatomical entity, catalytic activity and binding. In addition, KEGG analysis was performed (Figure 5C). Top 10 pathways of the different expressed proteins were listed in Table S2, with results showing the main pathways as carbohydrate metabolism, translation and amino acid metabolism.

### 3.6. Combined Analysis of Transcriptomics and Proteomics

**Figure 6:**
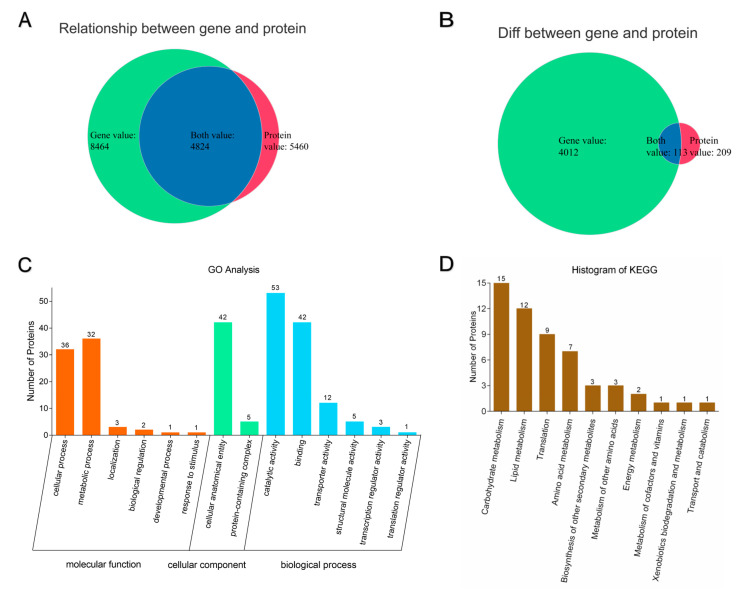
Combined analysis of transcriptomics and proteomics. The value of gene expression is significantly (*p* < 0.05) differentially expressed (>2-fold), value of protein expression is significantly (*p* < 0.05) differentially expressed (>1.5-fold) between two groups. (**A**) Venn of relationship between genes and proteins (**B**) Venn of matched differentially expressed genes and proteins (**C**) GO categorization of matched differentially expressed genes and proteins (**D**) Top 10 KEGG pathways of matched differentially expressed genes and proteins.

We identified 8464 genes at 8 h in transcriptomics, and 5460 proteins at 8 h in proteomics. Therefore, we investigated the relationship between these genes and proteins. Results showed that there were 4824 genes and proteins matched (Figure 6A), which means these genes could translate to the corresponding proteins. In addition, we attempted to match the 4 012 DEGs and 209 different expressed proteins, and 113 genes and proteins were matched (Figure 6B). Furthermore, GO and KEGG analysis were performed to identify these 113 matched genes and proteins. GO analysis revealed that the dominant terms were cellular process, metabolic process, cellular anatomical entity, catalytic activity and binding (Figure 6C), while KEGG analysis revealed that the pathways were enriched in carbohydrate metabolism, translation and amino acid metabolism. These results were similar to the above transcriptomics and proteomics results. The tree heatmap of combined KEGG analysis is shown in Figure S7.

## 4. Discussion

Our results demonstrated that the expression of numerous genes changed under low a_w_. Also, the KEGG pathway and fold change of major DEGs were shown in Table S3. For fungi, carbon is one of the most vital nutrients [33]. A study of *A. niger* [34] revealed that germination is initiated by mobilization of carbon stores. Our study proved that by changing a_w_, carbon metabolism was affected to a great extent. Among these DEGs, eight were down-regulated and one was up-regulated, inferring that low a_w_ may reduce sucrose utilization. *AFLA_053390* was described to be GPI-anchored cell wall beta-1,3-endoglucanase EglC, which was significantly down-regulated in our transcriptomic and down-regulated (no significance) in proteomic data in 0.90 a_w_ group. GPI-anchored cell wall proteins acted as a scaffold to support the cell wall in *yeast* [35]. The down-regulation of this protein may concern the cell wall folded phenotype in our SEM. Our transcriptomics and real-time quantitative PCR data in a protein phosphatase gene (*AFLA_050960*) were up-regulated in 0.90 a_w_ group, which was consistent with an earlier study [36], but in proteomic data the protein phosphatase showed no change, which indicated that some other progress may happen to balance the expression of the protein to maintain the steady state. In our data, compared to the 0.98 a_w_ group, *creA* was significantly up-regulated in 4 h, while it was significantly down-regulated in 8 h and 12 h (Figure S8). This phenomenon revealed that low a_w_ could affect conidia germination by affecting the expression of *creA*, and *creA* may play different roles in early conidia germination. In fact, carbon catabolite repression (CCR) has been widely studied [37] in carbon metabolism. The mechanism contains a group of genes, with *creA* being the major factor [38]. A study [39] revealed that *creA* is important in the morphology, pathogenicity and secondary metabolite production of *A. flavus*. On the other hand, glycolysis and the citric acid cycle (TCA cycle) are both important pathways in fungi. We found that low a_w_ also affected these two pathways, with three genes regulated in both glycolysis and the TCA cycle, which revealed that low a_w_ may change the ability of *A. flavus* to use carbon sources. Studies on *A. nidulans* [21] and *A. flavus* [40] revealed that these pathways are important in carbon metabolism and conidia germination. In TCA cycle, *AFLA_018850* was up-regulated in 0.90 a_w_ group; this gene is related to isocitrate dehydrogenase and is reported [41] to control metabolic flux through the citric acid cycle in a photo-switchable *yeast*, which indicated that the metabolism of citric acid cycle may be more severe under low a_w_.

Amino acid metabolism is an essential part of conidia germination. Germination of *A. niger* [34] has been found to be related to L-amino acids. Among the pathways related to amino acid metabolism in our data, the dominant pathways were concerned with arginine. Our results showed that *Clr4* was significantly down-regulated in 0.90 a_w_ group. Disruption of *Clr4* was reported to result in growth abnormalities [42]. In addition, our study revealed that the SAM protein was an important protein, and KEGG analysis showed that SAM protein was related to arginine metabolism. The expression of SAM protein was significantly down-regulated in 0.90 a_w_ group. A previous study [43] showed that *RmtA* targets the amino-terminal tails of arginine 3. Moreover, a study of *A. flavus* [44] revealed that *RmtA* is a putative arginine methyltransferase gene that could regulate conidia and sclerotia production, and also demonstrated that all *RmtA* homologs analyzed contained the SAM binding domain. Therefore, we inferred that low a_w_ may affect the metabolism of arginine through regulating *RmtA*, which finally resulted in a change in conidia germination. In addition, five genes were significantly regulated in tryptophan metabolism, revealing that low a_w_ has an impact on tryptophan metabolism. Thus, the connection between the effect of low a_w_ on tryptophan metabolism and conidia germination should be further studied. However, there are only a few studies that have investigated the relationship between tryptophan and *A. flavus*. It was reported [45] that tryptophan is connected to indole precursors in fungi. A study [46] demonstrated that tryptophan has an effect on aflatoxin biosynthesis and regulation in *A. flavus*. Moreover, our data showed that several genes changed in lysine biosynthesis and degradation by low a_w_. Lysine was reported [47] to cause repression of diaminopimelate decarboxylase synthesis in conidia germination. In *A. flavus* [48], lysine succinylation was speculated to be a mechanism that regulates aflatoxin production. Interestingly, our study also revealed changes in the TCA cycle and succinyl-CoA, resulting in the assumption that low a_w_ may down-regulate the succinylation of lysine. This may affect progress in the TCA cycle, which finally caused a decrease in conidia -germinating rate.

Translation pathways were also dominant pathways in our data. Our data in the translation pathways showed that by the effect of low a_w_, most genes were up-regulated compared to the 0.98 a_w_ group, with only one gene down-regulated. In fact, comparative transcriptomics analysis was performed in three different *Aspergillus* to reveal the mechanism of germination, and our study also found a variety of ribosome-related genes that were significantly enriched. In *A. fumigatus* [49], it was demonstrated that impaired ribosome biogenesis affects morphogenesis and nuclear duplication. In addition, in *A. flavus* [50], it was revealed that ribosomes are connected to aflatoxin production. We assumed that the up-regulation of these genes may have a positive effect to combine with more rRNA to form more ribosomes, which could help resist the down-regulation of a_w_. In addition, our data showed an increase in expression in RPL11 in 0.90 a_w_ group. A report [51] revealed that the ribosomal protein L11 induced cell apoptosis, which indicated that low a_w_ could result in cell apoptosis in early conidia germination. Moreover, the RNA transport pathway was affected, with eight genes significantly changed. Among these genes, we found low a_w_ affected translation initiation factors (eIFs) the most. In our data, eIF4E and eIF4B proteins were down-regulated in 0.90 a_w_ group. An earlier study [52] revealed that disruption of *eIF4E* contributed to growth retardation, which was consistent with our result that the down-regulation of eIF4E proteins caused a decrease in conidia germination rate. Another study [53] demonstrated that *eIF4B* controlled survival and proliferation, which verified our study. These results showed that one important way low a_w_ influenced the conidia germination of *A. flavus* was affecting ribosome biogenesis. However, the mechanism for how low a_w_ influences ribosomes to regulate conidia germination should be further studied.

## 5. Conclusions

In general, a_w_ could affect many aspects of germination of *A. flavus*. The morphology showed obvious changes in the size of the spore, germination rate, shape of fungi and membrane change. Transcriptome and proteome revealed that low a_w_ influenced *creA*, *TreB* in carbohydrate metabolism, *Clr4*, *RmtA* in amino acid metabolism, *RPL37, RPL3* in translation and *Hog1* and *Ste3* in the MAPK pathway, which demonstrated that low a_w_ caused a global change in *A. flavus* germination. In particular, we found that low a_w_ affects ribosome synthesis to a great extent, which indicated that ribosome synthesis may be the most important target pathway affected by change of a_w_.

## Data Availability

The sequencing data generated in this study have been deposited in NCBI’s Short Read Archive database (SRA, http://www.ncbi.nlm.nih.gov/Traces/sra_sub/sub.cgi) (accessed on 15 December 2020) and are accessible through SRA series accession number PRJNA694338. The mass spectrometry proteomics data have been deposited to the ProteomicsXchange Consortium (http://proteomecentral.proteomexchange.org/cgi/GetDataset?ID=PXD023808) (accessed on 18 December 2020) via the iProX partner repository [54] with the dataset identifier PXD023808.

## Supplementary Materials

The following supporting information can be downloaded at: https://www.mdpi.com/article/10.3390/microorganisms10091744/s1, Figure S1. Conidia germination rate in different water activity and time period. The means and standard errors of duplicate samples have been plotted (n = 6). Mean values without a common letter (a, b, c, d) were significantly different (*p* < 0.05). Figure S2. Principal component analysis (PCA) of transcriptome. The principal component PC1 could interpret 69.44% intergroup differences and principal component PC2 could interpret 11.74% intergroup difference, PC2 is orthogonal to PC1 (*n* = 3). − (0.98 a_w_) and + (0.90 a_w_) represented two different a_w_. Figure S3. Length distribution of assembled unigenes of transcriptome data. The length of unigenes ranged from 0 to over 1800 bp. Figure S4. Principal component analysis (PCA) of proteome. The principal component PC1 could interpret 52.40% intergroup differences and principal component PC2 could interpret 19.00% intergroup difference, PC2 is orthogonal to PC1 (*n* = 3). − (0.98 a_w_) and + (0.90 a_w_) represented two different a_w_. Figure S5. Peptide length distribution. This figure showed the number of different length of peptides. X-axis showed the peptide length, while Y-axis showed the corresponding peptide number. Figure S6. This figure showed the coverage distribution of identified proteins. Each sector represented the proportion of a coverage range. The larger the sector area was, the more proteins were covered in the range. The number outside the sector represented coverage range and proportion of proteins distributed in this range. Figure S7. Each column in the graph represents a protein or gene, and each row represents a KEGG pathway. The color in the graph represents the enrichment of proteins and genes in the pathway (The smaller the *p*-value is, the higher the enrichment degree is). The closer the two branches are, the closer their enrichment degree is. Figure S8. Real-time quantitative PCR of regulated genes mentioned in discussion. − (0.98 a_w_) and + (0.90 a_w_) represented two different a_w_. The means and standard errors of duplicate samples have been plotted (n = 6). Mean values with * were significantly different (*p* < 0.05). Table S1. Primer information. Table S2. Description of top 10 up-regulated proteins and down-regulated proteins in proteome. Table S3. The KEGG pathway and fold change of major DEGs.

## Appendix A

### Appendix A.1. Microscopy

#### Scanning Electron Microscope

Fresh spores should be selected to minimize mechanical damage such as pulling, contusion and extrusion. Wash spores with PBS gently to remove the impurity substance. The washed spores are immediately fixed by 2.5% glutaraldehyde for 2 h at room temperature, then transferred into 4 °C for preservation and transportation. Then wash sproes with 0.1 M PB (pH 7.4) for 3 times, 15 min each. Then transfer tissue blocks into 1% OsO_4_ in 0.1 M PB (pH 7.4) for 1–2 h at room temperature. After that, wash tissue blocks in 0.1 M PB (pH 7.4) for 3 times, 15 min each. Then gradient elution with ethanol (concentration of 30–100%) and dry samples with Critical Point Dryer. Specimens are attached to metallic stubs using carbon stickers and sputter-coated with gold for 30 s. Observe and take images with scanning electron microscope.

### Appendix A.2. RNA-Seq Analysis

#### Appendix A.2.1. RNA Extraction, cDNA Library Preparation, and SEQUENCING

Total RNA was extracted from the tissue using TRIzol^®^ Reagent (Plant RNA Purification Reagent for plant tissue) according the manufacturer’s instructions (Invitrogen) and genomic DNA was removed using DNase I (TaKara). Then RNA quality was determined by 2100 Bioanalyser (Agilent, Santa Clara, CA, USA) and quantified using the ND-2000 (NanoDrop Technologies, Wilmington, DE, USA). Only high-quality RNA sample (OD260/280 = 1.8~2.2, OD260/230 ≥ 2.0, RIN ≥ 6.5, 28S:18S ≥ 1.0, >1 μg) was used to construct sequencing library.

RNA-seq transcriptome library was prepared following TruSeqTM RNA sample preparation Kit from Illumina (San Diego, CA, USA) using 1μg of total RNA. Shortly, messenger RNA was isolated according to polyA selection method by oligo(dT) beads and then fragmented by fragmentation buffer firstly. Secondly double-stranded cDNA was synthesized using a SuperScript double-stranded cDNA synthesis kit (Invitrogen, CA) with random hexamer primers (Illumina). Then the synthesized cDNA was subjected to end-repair, phosphorylation and ‘A’ base addition according to Illumina’s library construction protocol. Libraries were size selected for cDNA target fragments of 300 bp on 2% Low Range Ultra Agarose followed by PCR amplified using Phusion DNA polymerase (NEB) for 15 PCR cycles. After quantified by TBS380, paired-end RNA-seq sequencing library was sequenced with the Illumina HiSeq xten/NovaSeq 6000 sequencers (2 × 150 bp read length).

#### Appendix A.2.2. Read Mapping, Differential Expression Analysis and Functional Enrichment

The raw paired end reads were trimmed and quality controlled by SeqPrep (https://github.com-/jstjohn/SeqPrep) (accessed on 2 July 2020) and Sickle (https://github.com/n-ajoshi/sickle) (accessed on 2 July 2020) with default parameters. Then clean reads were separately aligned to reference genome with orientation mode using HISAT2 (version 2.1.0) (http://ccb.jh-u.edu/software-/hisat2/index.shtmL) (accessed on 8 July 2020) [25] software. The mapped reads of each sample were assembled by StringTie (https://ccb.jhu.edu/software/stringtie-/index.shtmL?t=example) (accessed on 10 July 2020) in a reference-based approach [26].

To identify DEGs (differential expression genes) between two different samples, the expression level of each transcript was calculated according to the transcripts per million reads (TPM) method. RSEM (version 1.3.1) (http://deweylab.biostat.wisc.edu/rsem/) (accessed on 15 July 2020) [55] was used to quantify gene abundances. Essentially, differential expression analysis was performed using the DESeq2 (version 1.38.0) [56] with *p* value ≤ 0.05 were considered to be significantly different expressed genes. In addition, functional-enrichment analysis including GO and KEGG were performed to identify which DEGs were significantly enriched in GO terms and metabolic pathways at Bonferroni-corrected *p*-value ≤ 0.05 compared with the whole-transcriptome background. GO functional enrichment (version 2020.0628) and KEGG pathway analysis (version 2020.07) were carried out by Goatools (version 0.6.5) (https://github.com/tanghaibao/Goatools) (accessed on 25 July 2020) and KOBAS (version 2.1.1) (http://kobas.cbi.pk-u.edu.cn/home.do) (accessed on 25 July 2020) [29].

#### Appendix A.2.3. Alternative Splice Events Identification

All the alternative splice events that occurred in our sample were identified by using recently releases program rMATS (version 3.2.5) (http://rnaseq-mats.sour-ceforge.net/index.htmL) (accessed on 15 July 2020) [57]. Only the isoforms that were similar to the reference or comprised novel splice junctions were considered, and the splicing differences were detected as exon inclusion, exclusion, alternative 5′, 3′, and intron retention events.

### Appendix A.3. TMT Labeling Analysis

#### Appendix A.3.1. Total Protein Extraction, Protein Digestion and TMT Labeling

Total protein was extracted from fungal samples by using a urea lysis buffer (7 M urea, 2 M thiourea, and 1% SDS) with a protease inhibitor. Protein concentrations were detected with a BCA Protein Assay Kit (Pierce, Thermo, Waltham, MA, USA). Following reduction, cysteine alkylation, and digestion, samples were labeled with iTRAQ Reagents (Applied Biosystems, 4390812, Walthamm, MA, USA) according to the manufacturer’s instructions. After being desalted with a C18 solid-phase extraction, peptides were used for Nano Liquid Chromatography–Mass Spectrometry/Mass Spectrometry (LC-MS/MS) analysis [31].

Protein digestion was performed according to the standard procedure and the resulting peptide mixture was labeled using the 10-plex TMT reagent (Thermo fisher, Art.No.90111) according to the manufacturer’s instructions. Briefly, total protein (100 μg) taken from each sample was mixed with 100 mL of the lysate. TCEP (10 mM) was added and then it was stored at 37 °C. 60 min later, iodoacetamide (40 mM) was added and stored in dark at room temperature for 40 min.

Sixfold volumes of cold acetone were added to precipitate protein at –20 °C for 4 h. After centrifugation at 10,000× *g* at 4 °C for 20 min, the pellet was re-suspended with 100 µL 50 mM riethylammonium bicarbonate (TEAB) buffer. Trypsin was added at 1:50 trypsin-to-protein mass ratio and incubated at 37 °C overnight. Then, one unit of TMT reagent were thawed and reconstituted in 50 µL acetonitrile, after tagging for 2 h at room temperature, hydroxylamine was added to react for 15 min at room temperature. In our study, the samples were labeled as (0.98-1)-113, (0.98-2)-114 and (0.98-3)-115, (0.95-1)-116, (0.95-2)-117 and (0.95-3)-118.Finally, all samples were pooled, desalted and vacuum-dried.

#### Appendix A.3.2. LC-MS/MS Analysis

Labeled peptides were analyzed by online nano flow liquid chromatography tandem mass spectrometry performed on an 9RKFSG2_NCS-3500R system (Thermo, USA) connected to a Q Exactive Plus quadrupole orbitrap mass spectrometer (Thermo, USA) through a nano electrospray ion source. Briefly, the C18-reversed phase column (75 μm × 25 cm, Thermo, USA) as equilibrated with solvent A (A:2% formic acid with 0.1% formic acid) and solvent B (B: 80% ACN with 0.1% formic acid). The peptides were eluted using the following gradient: 0–4 min, 0%–5% B; 4–66 min, 5%−23% B; 66–80 min, 23%−29% B; 80−89 min, 29%−38% B; 89–91 min, 38–48% B; 91–92 min, 48–100% B; 92–105 min, 100% B; 105–106 min, 100–0% B) at a flow rate of 300nL/min. The Q Exactive Plus was operated in the data-dependent acquisition mode (DDA) to automatically switch between full scan MS and MS/MS acquisition. The survey of full scan MS spectra (m/z 350–1300) was acquired in the Orbitrap with 70000 resolutions. The automatic gain control (AGC) target at 3e6 and the maximum fill time was 20 ms. Then the top 20 most intense precursor ions were selected into collision cell for fragmentation by higher-energy collision dissociation (HCD). The MS/MS resolution was set at 35000 (at m/z 100), the automatic gain control (AGC) target at 1e5, the maximum fill time at 50 ms, and dynamic exclusion was 18 s.

#### Appendix A.3.3. Protein Identification

The RAW data files were analyzed using Proteome Discoverer (Thermo Scientific, Version 2.2) Against *Aspergillus. flavus* database.

(Aspergillus_flavus.JCVI-afl1-v2.0.pep.unique.fa.MJ20200708085.fasta). The MS/MS search criteria were as follows: Mass tolerance of 10 ppm for MS and 0.02 Da for MS/MS Tolerance, trypsin as the enzyme with 2 missed cleavage allowed, carbamido methylation of cysteine and the TMT of N- terminus and lysine side chains of peptides as fixed modification, and methionine oxidation as dynamic modifications, respectively. False discovery rate (FDR) of peptide identification was set as FDR ≤ 0.01. A minimum of one unique peptide identification was used to support protein identification.

#### Appendix A.3.4. Statistical Analysis

Annotation of all identified proteins was performed Gene Ontology (GO; http://www.blast2go.com/b2ghome; http://geneontology.org/) (accessed on 3 October 2020) and KEGG pathway (http://www.genome.jp/kegg/) (accessed on 3 October 2020). PCA analysis was perfomed by sklearn in python, and heatmap waz performed by pheatmap (version 1.0.12).
