# Supplementary material for: Effect of Water Activity on Conidia Germination in Aspergillus flavus
Source: Microorganisms. 2022 Aug 29;10(9):1744. doi: 10.3390/microorganisms10091744 (PMC9504883; doi:10.3390/microorganisms10091744)
Supplement: Supplementary file 1 [file microorganisms-10-01744-s001.zip › microorganisms-1856907-supplementary.pdf]

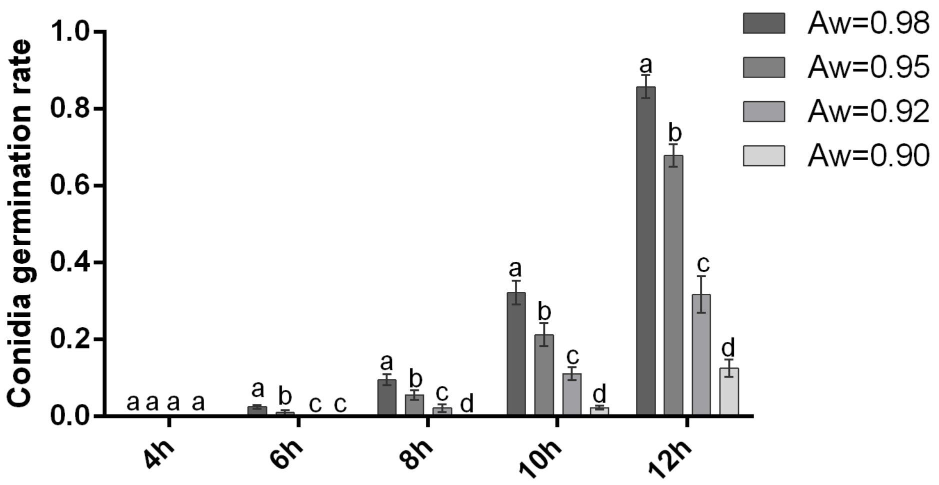

Figure S1. Conidia germination rate in different water activity and time period. The means and standard errors of duplicate samples have been plotted (n=6). Mean values without a common letter (a, b, c, d) were significantly different ($p < 0.05$).

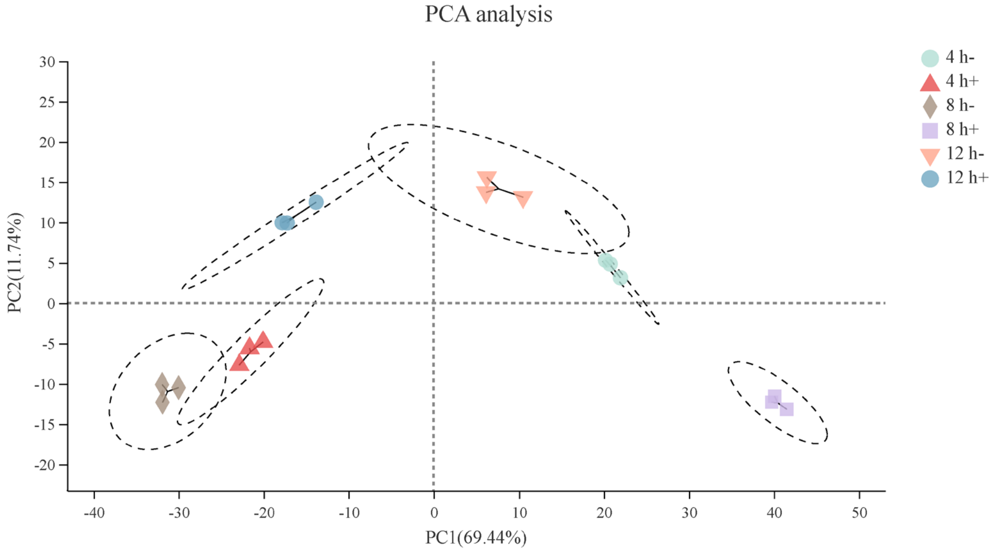

Figure S2. Principal component analysis (PCA) of transcriptome. The principal component PC1 could interpret 69.44 % intergroup differences and principal component PC2 could interpret 11.74 % intergroup difference, PC2 is orthogonal to PC1 (n = 3). - (0.98 a_w) and + (0.90 a_w) represented two different a_w.

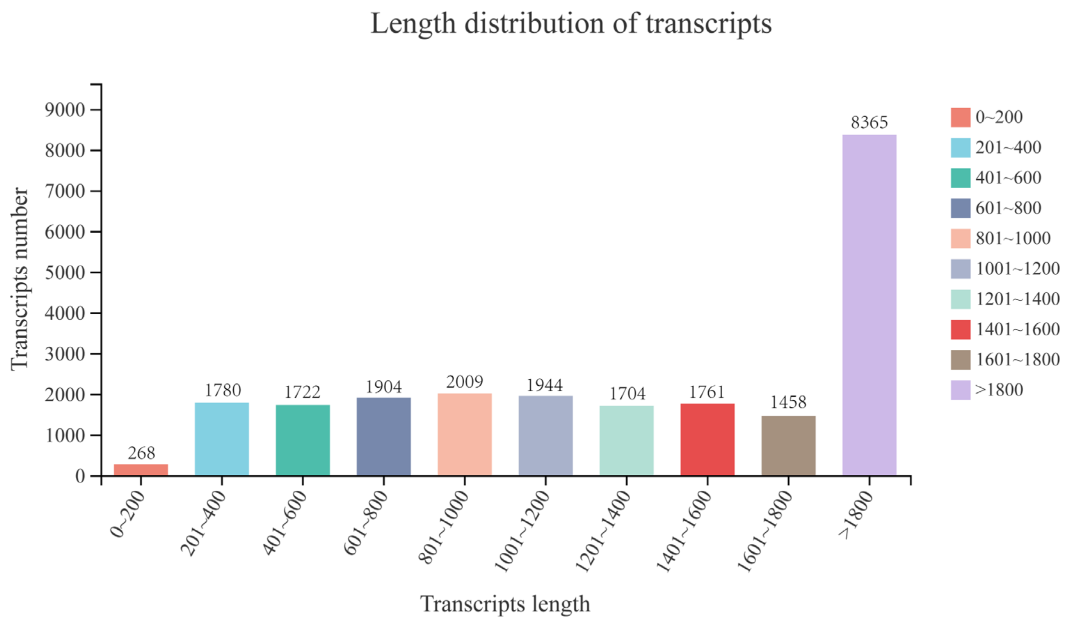

Figure S3. Length distribution of assembled unigenes of transcriptome data. The length of unigenes ranged from 0 to over 1800 bp.

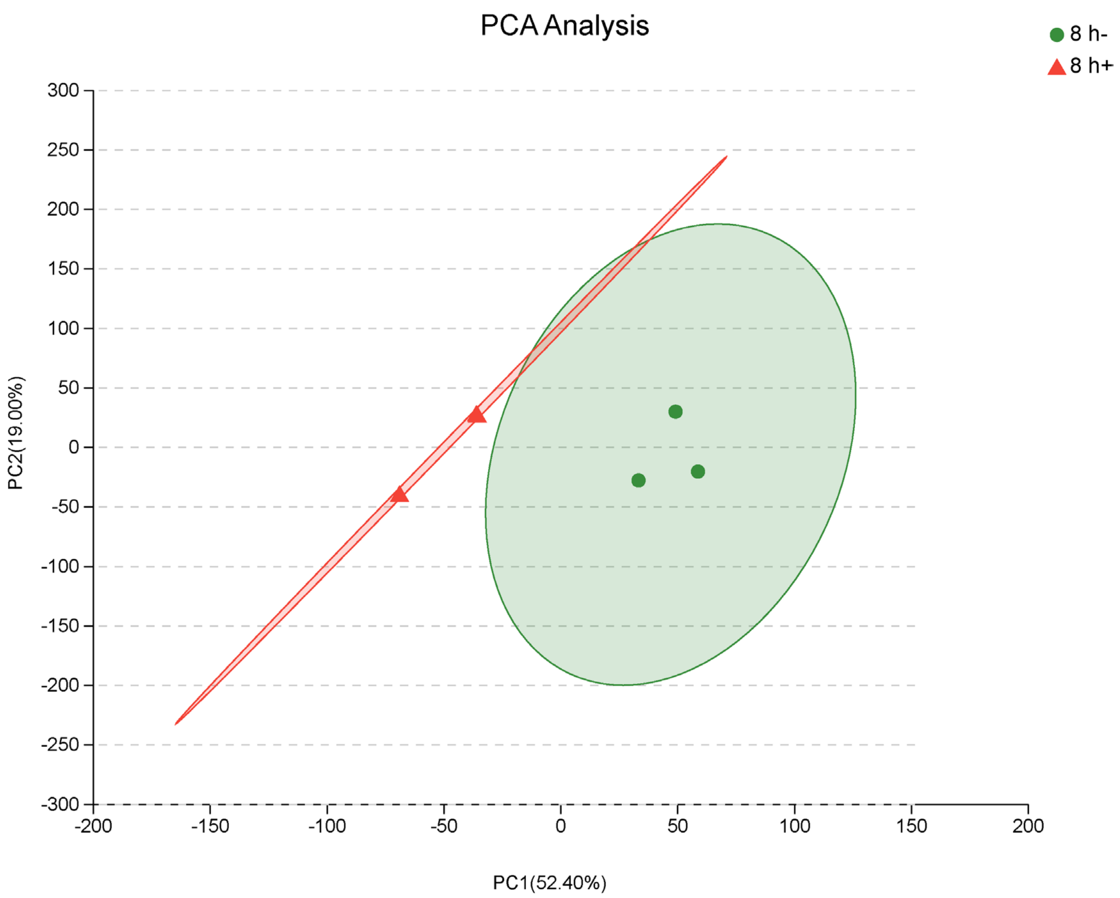

Figure S4. Principal component analysis (PCA) of proteome. The principal component PC1 could interpret 52.40 % intergroup differences and principal component PC2 could interpret 19.00 % intergroup difference, PC2 is orthogonal to PC1 (n = 3). - (0.98 aw) and + (0.90 aw) represented two different a_w.

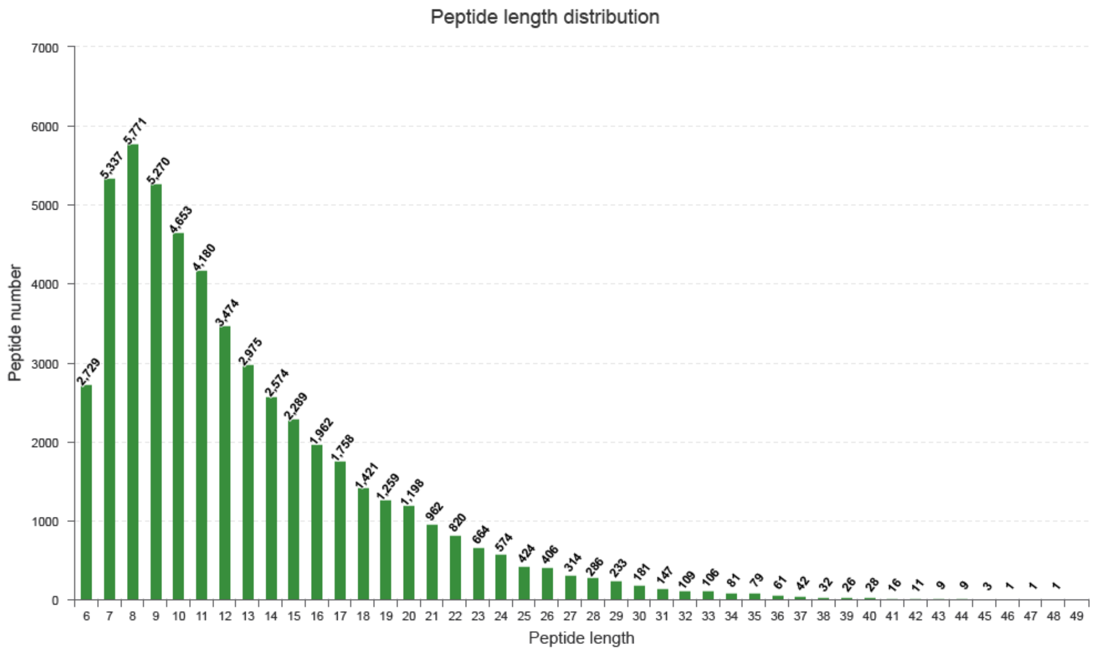

Figure S5. Peptide length distribution. This figure showed the number of different length of peptides. X-axis showed the peptide length, while Y-axis showed the corresponding peptide number.

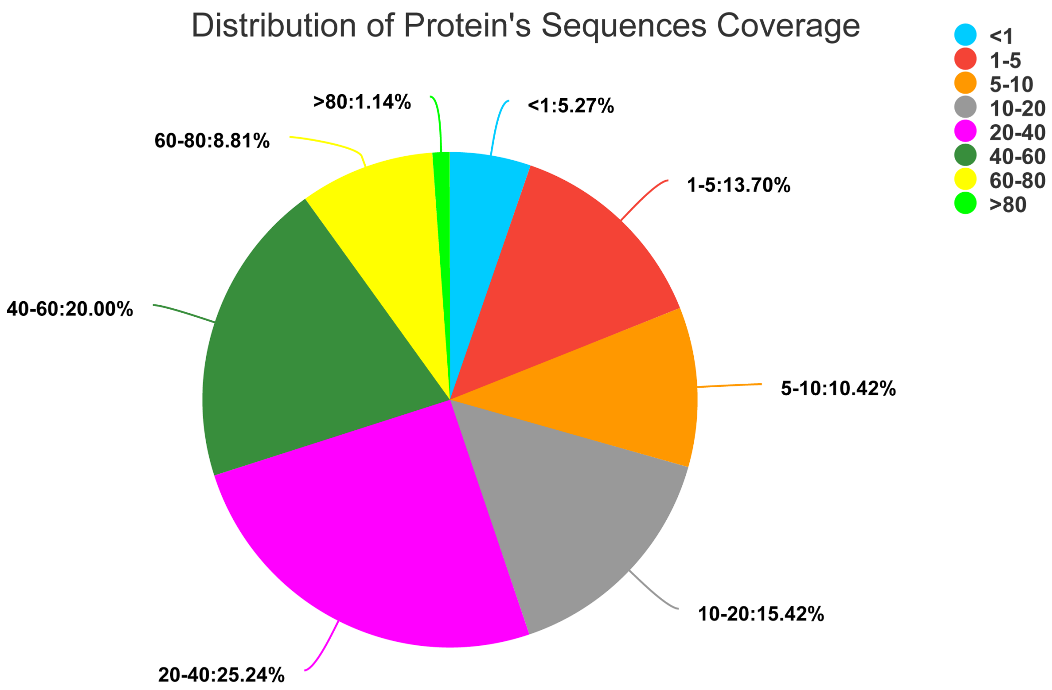

Figure S6. This figure showed the coverage distribution of identified proteins. Each sector represented the proportion of a coverage range. The larger the sector area was, the more proteins were covered in the range. The number outside the sector represented coverage range and proportion of proteins distributed in this range.

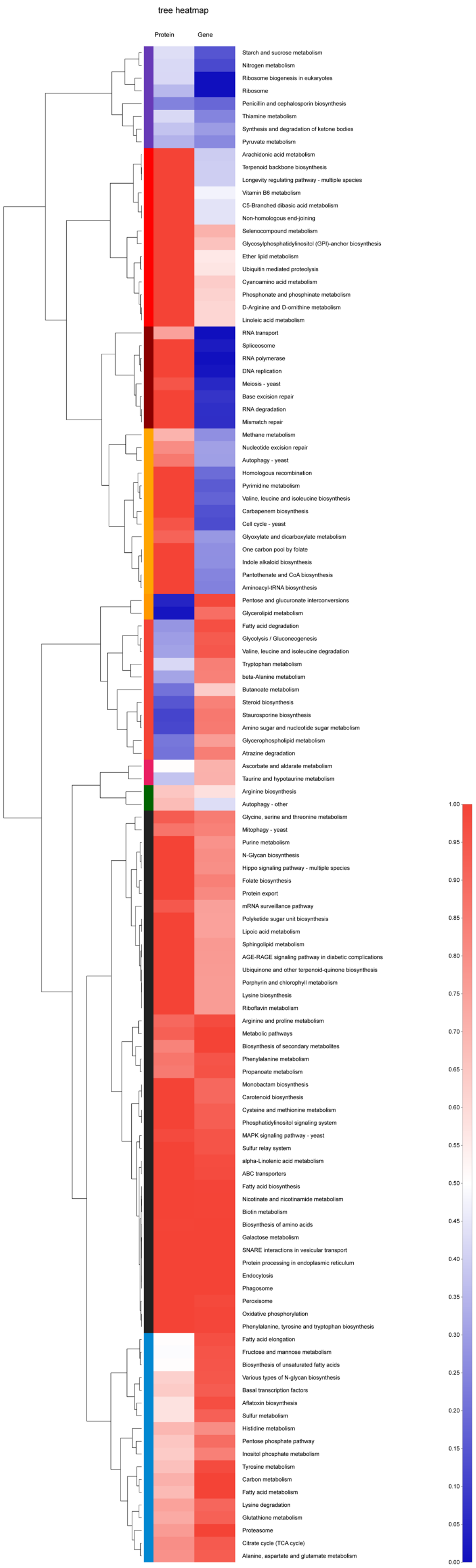
tree heatmap
Protein
Gene
Starch and sucrose metabolism
Nitrogen metabolism
Ribosome biogenesis in eukaryotes
Ribosome
Penicillin and cephalosporin biosynthesis
Thiamine metabolism
Synthesis and degradation of ketone bodies
Pyruvate metabolism
Arachidonic acid metabolism
Terpenoid backbone biosynthesis
Longevity regulating pathway - multiple species
Vitamin B6 metabolism
C5-Branched dibasic acid metabolism
Non-homologous end-joining
Selenocompound metabolism
Glycosylphosphatidylinositol (GPI)-anchor biosynthesis
Ether lipid metabolism
Ubiquitin mediated proteolysis
Cyanoamino acid metabolism
Phosphonate and phosphinate metabolism
D-Arginine and D-ornithine metabolism
Linoleic acid metabolism
RNA transport
Spliceosome
RNA polymerase
DNA replication
Meiosis - yeast
Base excision repair
RNA degradation
Mismatch repair
Methane metabolism
Nucleotide excision repair
Autophagy - yeast
Homologous recombination
Pyrimidine metabolism
Valine, leucine and isoleucine biosynthesis
Carbapenem biosynthesis
Cell cycle - yeast
Glyoxylate and dicarboxylate metabolism
One carbon pool by folate
Indole alkaloid biosynthesis
Pantothenate and CoA biosynthesis
Aminoacyl-tRNA biosynthesis
Pentose and glucuronate interconversions
Glycerolipid metabolism
Fatty acid degradation
Glycolysis / Gluconeogenesis
Valine, leucine and isoleucine degradation
Tryptophan metabolism
beta-Alanine metabolism
Butanoate metabolism
Steroid biosynthesis
Staurosporine biosynthesis
Amino sugar and nucleotide sugar metabolism
Glycerophospholipid metabolism
Atrazine degradation
Ascorbate and aldarate metabolism
Taurine and hypotaurine metabolism
Arginine biosynthesis
Autophagy - other
Glycine, serine and threonine metabolism
Mitophagy - yeast
Purine metabolism
N-Glycan biosynthesis
Hippo signaling pathway - multiple species
Folate biosynthesis
Protein export
mRNA surveillance pathway
Polyketide sugar unit biosynthesis
Lipoic acid metabolism
Sphingolipid metabolism
AGE-RAGE signaling pathway in diabetic complications
Ubiquinone and other terpenoid-quinone biosynthesis
Porphyrin and chlorophyll metabolism
Lysine biosynthesis
Riboflavin metabolism
Arginine and proline metabolism
Metabolic pathways
Biosynthesis of secondary metabolites
Phenylalanine metabolism
Propanoate metabolism
Monobactam biosynthesis
Carotenoid biosynthesis
Cysteine and methionine metabolism
Phosphatidylinositol signaling system
MAPK signaling pathway - yeast
Sulfur relay system
alpha-Linolenic acid metabolism
ABC transporters
Fatty acid biosynthesis
Nicotinate and nicotinamide metabolism
Biotin metabolism
Biosynthesis of amino acids
Galactose metabolism
SNARE interactions in vesicular transport
Protein processing in endoplasmic reticulum
Endocytosis
Phagosome
Peroxisome
Oxidative phosphorylation
Phenylalanine, tyrosine and tryptophan biosynthesis
Fatty acid elongation
Fructose and mannose metabolism
Biosynthesis of unsaturated fatty acids
Various types of N-glycan biosynthesis
Basal transcription factors
Aflatoxin biosynthesis
Sulfur metabolism
Histidine metabolism
Pentose phosphate pathway
Inositol phosphate metabolism
Tyrosine metabolism
Carbon metabolism
Fatty acid metabolism
Lysine degradation
Glutathione metabolism
Proteasome
Citrate cycle (TCA cycle)
Alanine, aspartate and glutamate metabolism
1.00
0.95
0.90
0.85
0.80
0.75
0.70
0.65
0.60
0.55
0.50
0.45
0.40
0.35
0.30
0.25
0.20
0.15
0.10
0.05
0.00

Figure S7. Each column in the graph represents a protein or gene, and each row represents a KEGG pathway. The color in the graph represents the enrichment of proteins and genes in the pathway (The smaller the *p*-value is, the higher the enrichment degree is). The closer the two branches are, the closer their enrichment degree is.

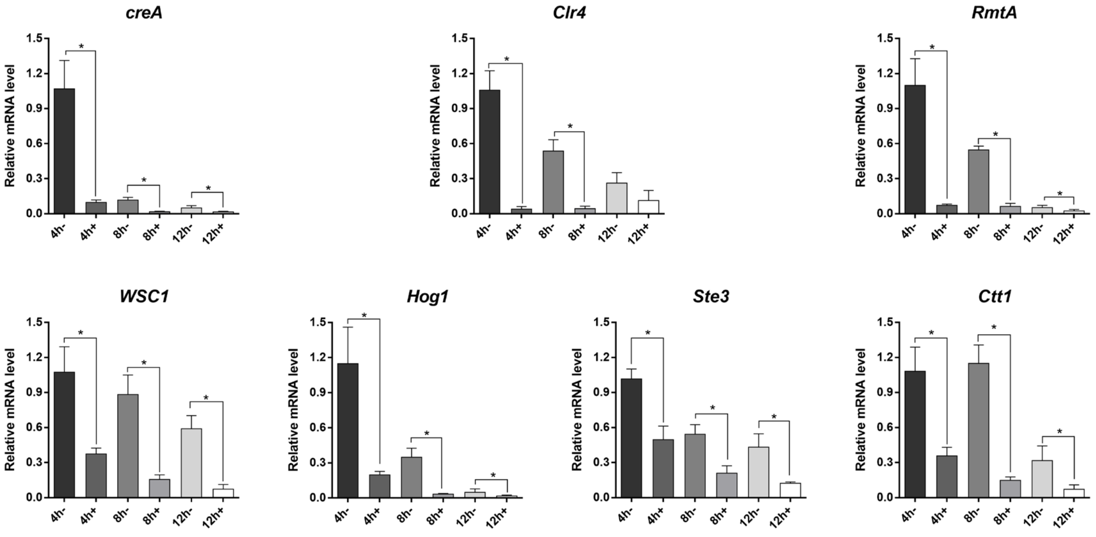

Figure S8. Real-time quantitative PCR of regulated genes mentioned in discussion. - (0.98 aw) and + (0.90 aw) represented two different aw. The means and standard errors of duplicate samples have been plotted (n = 6). Mean values with * were significantly different ($p < 0.05$).

Table S1. Primer information.

Gene name	Primer direction	Sequences (5′to 3′)	PCR (bp)	Accession
AFLA_050960	Forward<br>Reverse	AACGGTATTCGTTCGACTGG<br>CTTGGAGAAAGCCAAACTCG	195	XM_002383181.1
AFLA_127350	Forward<br>Reverse	GATCAATCCGGATAGCCTCA<br>ATACGGTCGCCATCAAGAAG	186	XM_002381887.1
AFLA_058590	Forward<br>Reverse	CAAGAGTGGCATTGCGTCTA<br>TTCAAGCTTGGCTACCGAGT	169	XM_002378562.1
AFLA_052430	Forward<br>Reverse	TCGAGGATGTAGGACCCAAG<br>GACTGGGTTCTCGCTCAGAC	179	XM_002383328.1
AFLA_044550	Forward<br>Reverse	GGCTTGGACTACCGTGTGAT<br>TGACATGCTGGAACTTGCTC	235	XM_002377876.1
AFLA_033490	Forward<br>Reverse	TGGGAAAGAGGAGGAAGGTT<br>AATGCGACGGTCCGTATTAG	250	XM_002374818.1
AFLA_068470	Forward<br>Reverse	GTCGTCGATGGACAGAGGTT<br>TGTGCCAGGGTTGTAATCAA	183	XM_002380365.1
AFLA_106820	Forward<br>Reverse	GACATGGAGATAGCCCGAGA<br>CAATGCGATTTCCCACTTTT	163	XM_002376512.1
AFLA_100940	Forward<br>Reverse	ACGAAGGGTACCTCCAGCTT<br>GCCTTCTCGCTCCAGTTGTA	279	XM_002383925.1
AFLA_044520	Forward<br>Reverse	TCTTCCGTCGGTATTGATGC<br>CATGTCCTCATCGGACTCCT	228	XM_002377873.1
AFLA_134740	Forward<br>Reverse	CCATCTTCGAGAAGGACGAG<br>GTTGCAAGAGGTACGGTGGT	218	XM_002379446.1
AFLA_025760	Forward<br>Reverse	TGTCAATTTCTTCGCAGGTC<br>CAGCGTCAATGTGCTGATCT	194	XM_002374045.1
AFLA_052510	Forward<br>Reverse	CTTCTTCGATCGCTGGTTTC<br>TGAAGTGTTCGAGCGAGATG	172	XM_002383336.1
AFLA_078340	Forward<br>Reverse	ACAGCGCCTTAATTCACCAC<br>TCGAAGTCGCAGTGTTCATC	157	XM_002372710.1
AFLA_034380	Forward<br>Reverse	TGAGACTCTCGCCCATTTCT<br>CCCAGTCCAAGTTACCCTCA	195	XM_002374907.1
AFLA_061620	Forward<br>Reverse	CTTCTTCGATCGCTGGTTTC<br>TGAAGTGTTCGAGCGAGATG	172	XM_002378865.1
AFLA_134680	Forward<br>Reverse	CTCAGGTTGAGCGTGATGAA<br>GCTTGACGCGATGAGTGTAA	183	XM_002379440.1
AFLA_127370	Forward<br>Reverse	TCTCCGAGTGGATGGGTTAC<br>TCCCAGAATCCGATCTTGTC	172	XM_002381889.1

Table S2. Description of top 10 up-regulated proteins and down-regulated proteins in proteome

Protein	Description	Log_2FC ($0.90a_w$/ $0.98a_w$)
EED55101	phosphocarrier protein HPr, putative	1.825
EED57541	alcohol dehydrogenase, putative	1.509
EED54712	efflux pump antibiotic resistance protein, putative	1.330
EED53174	DUF636 domain protein	1.313
EED46333	thiol methyltransferase, putative	1.276
EED45930	zinc-binding alcohol dehydrogenase, putative	1.077
EED46660	proteasome regulatory particle subunit (RpnC), putative	1.076
EED48289	DUF427 domain protein	1.070
EED45335	C6 finger domain protein, putative	1.054
EED47731	sugar transporter, putative	1.009
EED49459	Polysaccharide lyase	-1.012
EED51091	cholestenol delta-isomerase, putative	-1.028
EED55906	telomere and ribosome associated protein Stm1, putative	-1.051
EED53678	nucleolar GTPase, putative	-1.087
EED47908	hypothetical protein AFLA_005500	-1.092
EED52263	NADH-cytochrome B5 reductase, putative	-1.115
EED53847	60S ribosomal protein L34	-1.183
EED58025	glutathione S-transferase, putative	-1.189
EED52815	alpha/beta hydrolase, putative	-1.283
EED51400	UPF0187 domain membrane protein	-1.293

Table S3. The KEGG pathway and fold change of major DEGs.

Pathway	Gene ID	Description	Log_2FC (4 h)	Log_2FC (8 h)	Log_2FC (12 h)
Carbohydrate metabolism					
Pentose phosphate pathway	AFLA_031900	phosphoketolase, putative	-1.11	-2.54	3.05
	AFLA_119950	fructose-bisphosphate aldolase, putative	-2.82	-1.19	-1.52
	AFLA_076630	ribose phosphate diphosphokinase Prs1, putative	1.15	1.69	1.10
Citrate cycle (TCA cycle)	AFLA_076690	conserved hypothetical protein	-2.53	-3.63	-1.60
	AFLA_018850	isocitrate dehydrogenase, NAD-dependent	1.01	1.13	1.27
	AFLA_048610	succinyl-CoA synthetase alpha subunit, putative	1.12	1.37	1.12
Glycolysis / Gluconeogenesis	AFLA_031570	pyruvate decarboxylase PdcA, putative	-4.43	-1.82	-1.04
	AFLA_076690	conserved hypothetical protein	-2.53	-3.63	-1.60
	AFLA_119950	fructose-bisphosphate aldolase, putative	-2.82	-1.19	-1.52
Pyruvate metabolism	AFLA_078380	acetyl-coA hydrolase Ach1, putative	-2.31	-1.61	-1.07
	AFLA_076690	conserved hypothetical protein	-2.53	-3.63	-1.60
	AFLA_031910	acetate kinase, putative	-1.31	-2.74	-1.01
	AFLA_003700	mitochondrial cytochrome b2, putative	-2.20	1.56	1.40
Glyoxylate and dicarboxylate metabolism	AFLA_123600	oxidoreductase, putative	1.71	-1.59	-1.19
	AFLA_034380	catalase, putative	-2.98	-1.61	1.52
	AFLA_058590	dihydrodipicolinate synthetase family protein	-1.93	-1.97	-1.40
Amino sugar and nucleotide sugar metabolism	AFLA_030230	DTDP-glucose 4-6-dehydratase, putative	-1.27	1.04	-1.45
	AFLA_127350	N-acetylglucosamine-phosphate mutase	1.05	1.04	1.37
	AFLA_031380	class V chitinase, putative	-1.50	-1.76	-2.49
	AFLA_031390	ubiquitin (UbiC), putative	2.39	2.82	1.68
Butanoate metabolism	AFLA_070820	3-hydroxymethyl-3-methylglutaryl-Coenzyme A lyase, putative	-2.58	-2.03	-1.56
	AFLA_032500	glyoxylate carboligase, putative	1.05	1.61	1.59
	AFLA_000930	acetolactate synthase, large subunit, putative	3.24	2.51	1.81
	AFLA_119710	hydroxymethylglutaryl-CoA synthase, putative	1.36	-1.30	-1.07
	AFLA_023490	alpha-amylase, putative	-1.77	-1.05	-1.76

Starch and sucrose metabolism	AFLA_026150	alpha-glucosidase AgdA, putative	-1.67	-3.44	-1.77
	AFLA_053390	GPI-anchored cell wall beta-1,3-endoglucanase EglC	2.26	-1.32	-1.48
	AFLA_051140	beta-glucosidase, putative	-1.03	-2.69	-1.33
	AFLA_123170	amylase, putative	1.92	2.10	2.30
	AFLA_052430	alpha,alpha-trehalase TreB/Nth1	-1.97	-2.28	-1.22
	AFLA_058600	beta-fructofuranosidase, putative	-2.47	-2.63	-1.25
	AFLA_081340	glycogen debranching enzyme Gdb1, putative	-1.75	-1.17	-2.18
	AFLA_018550	glycogen phosphorylase GlpV/Gph1, putative	-1.37	-2.37	-1.93
Inositol phosphate metabolism	AFLA_050960	protein phosphatase	1.01	1.48	1.49
Fructose and mannose metabolism	AFLA_119950	fructose-bisphosphate aldolase, putative	-2.82	-1.91	-1.52
	AFLA_093790	dihydroxyacetone kinase (DakA), putative	2.85	2.14	1.63
Galactose metabolism	AFLA_026150	alpha-glucosidase AgdA, putative	-1.67	-3.44	-1.77
	AFLA_123170	amylase, putative	1.92	2.10	2.30
	AFLA_058600	beta-fructofuranosidase, putative	-2.47	-2.63	-1.25
Propanoate metabolism	AFLA_117280	2-methylcitrate dehydratase (PrpD), putative	-1.76	-3.47	-2.91
	AFLA_048610	succinyl-CoA synthetase alpha subunit, putative	1.12	1.37	1.12
	AFLA_031910	acetate kinase, putative	-1.31	-2.74	-1.01
C5-Branched dibasic acid metabolism	AFLA_032500	glyoxylate carboligase, putative	1.05	1.61	1.59
	AFLA_000930	acetolactate synthase, large subunit, putative	3.24	2.51	1.81
Amino acid metabolism					
Lysine degradation	AFLA_068470	histone-lysine N-methyltransferase Clr4	-1.16	-1.47	-1.47
	AFLA_137280	sarcosine oxidase, putative	-1.62	-2.51	-1.00
	AFLA_004220	FAD dependent oxidoreductase, putative	1.28	-1.17	-2.20
Tyrosine metabolism	AFLA_125240	aromatic-L-amino-acid decarboxylase, putative	1.23	1.84	1.84
	AFLA_108370	tyrosinase, putative	4.61	3.08	1.44
Cysteine and methionine	AFLA_006490	S-adenosylmethionine decarboxylase proenzyme	1.51	1.92	1.60
Alanine, aspartate and	AFLA_108100	argininosuccinate synthase	2.01	1.31	2.25
	AFLA_044550	carbamoyl-phosphate synthase, large subunit	2.77	2.14	2.46

glutamate metabolism	AFLA_108960	bifunctional pyrimidine biosynthesis protein (PyrABCN), putative	1.48	2.05	2.32
	AFLA_099960	argininosuccinate lyase	1.69	1.27	2.00
Phenylalanine, tyrosine and tryptophan biosynthesis	AFLA_073290	phospho-2-dehydro-3-deoxyheptonate aldolase	1.44	1.67	1.72
	AFLA_017680	pentafunctional polypeptide (AroM), putative	1.34	1.17	1.48
Arginine biosynthesis	AFLA_108100	argininosuccinate synthase	2.01	1.31	2.25
	AFLA_099960	argininosuccinate lyase	1.69	1.27	2.00
Lysine biosynthesis	AFLA_087950	isocitrate dehydrogenase LysB	1.19	1.67	2.00
	AFLA_127840	aconitate hydratase, mitochondrial	1.18	1.12	1.91
Arginine and proline metabolism	AFLA_042490	DUF521 domain protein	1.31	1.89	1.37
	AFLA_106820	FAD dependent oxidoreductase superfamily	-1.97	-2.19	-2.03
	AFLA_035040	acetyltransferase, GNAT family, putative	1.20	2.08	2.04
	AFLA_006490	S-adenosylmethionine decarboxylase proenzyme	1.51	1.92	1.60
	AFLA_000540	amidase, putative	1.42	2.24	1.90
	AFLA_129920	agmatinase, putative	-1.03	-1.49	1.74
	AFLA_058590	dihydrodipicolinate synthetase family protein	-1.83	-1.97	-1.40
Glycine, serine and threonine metabolism	AFLA_106820	FAD dependent oxidoreductase superfamily	-1.97	-2.19	-2.03
	AFLA_053220	conserved hypothetical protein	1.81	-2.25	-1.71
	AFLA_137280	sarcosine oxidase, putative	-1.62	-2.51	-1.00
	AFLA_004220	FAD dependent oxidoreductase, putative	1.28	-1.17	-2.20
	AFLA_052530	phosphatidylserine synthase	1.49	1.73	1.32
Tryptophan metabolism	AFLA_125240	aromatic-L-amino-acid decarboxylase, putative	1.23	1.84	1.84
	AFLA_025850	kynureninase, putative	-1.69	-1.58	-2.08
	AFLA_033490	nitrilase	1.49	3.04	2.24
	AFLA_000540	amidase, putative	1.42	2.24	1.90
	AFLA_034380	catalase, putative	-2.98	-1.61	1.52
Valine, leucine and isoleucine degradation	AFLA_070820	3-hydroxymethyl-3-methylglutaryl-Coenzyme A lyase, putative	-2.58	-2.03	-1.56
	AFLA_119960	oxidoreductase, putative	-2.30	-5.12	-2.73
	AFLA_119710	hydroxymethylglutaryl-CoA synthase, putative	1.36	-1.30	-1.07
Valine, leucine and isoleucine biosynthesis	AFLA_032500	glyoxylate carboligase, putative	1.05	1.61	1.59
	AFLA_000930	acetolactate synthase, large subunit, putative	3.24	2.51	1.81

Phenylalanine metabolism	AFLA_125240	aromatic-L-amino-acid decarboxylase, putative	1.23	1.84	1.84
	AFLA_000540	amidase, putative	1.42	2.24	1.90
Translation					
Ribosome biogenesis in eukaryotes	AFLA_033570	SSU processome component Utp10, putative	1.84	1.96	1.62
	AFLA_020450	nucleolar protein nop5	1.14	2.32	2.10
	AFLA_080740	small nucleolar ribonucleoprotein complex subunit Dip2, putative	1.87	2.08	1.15
	AFLA_134410	nucleolar GTP-binding protein (Nog1), putative	1.38	1.75	1.45
	AFLA_005850	exonuclease Kem1, putative	-10.03	-1.03	-1.10
	AFLA_005820	small nucleolar ribonucleoprotein complex subunit (Pwp2), putative	1.33	2.62	2.16
	AFLA_016750	exonuclease, putative	1.19	2.16	1.17
	AFLA_029920	nucleolar ATPase Kre33, putative	1.39	2.35	2.40
	AFLA_112310	small nucleolar ribonucleoprotein complex subunit, putative	1.74	2.24	2.28
	AFLA_016990	fibrillarin	1.57	2.27	2.27
	AFLA_088980	snoRNA binding protein, putative	1.26	2.16	1.48
	AFLA_026620	small nucleolar ribonucleoprotein complex component (Utp5), putative	1.04	2.32	2.40
	AFLA_042550	small nucleolar ribonucleoprotein complex subunit Utp15, putative	1.68	2.21	1.57
	AFLA_028940	small nucleolar ribonucleoprotein complex subunit Utp14, putative	1.90	11.60	9.81
	AFLA_134990	midasin, putative	1.78	1.71	1.99
	AFLA_113720	ribosome biogenesis (Nop4), putative	1.35	2.55	2.32
	AFLA_030720	RNA exonuclease Rex2, putative	-1.00	1.51	1.20
	AFLA_130080	small nucleolar ribonucleoprotein complex subunit, putative	1.04	1.61	1.12
Ribosome	AFLA_029450	Ribosomal L18ae protein family	1.27	1.67	1.56
	AFLA_044170	40S ribosomal protein S11	1.49	2.04	2.00
	AFLA_115110	60S ribosomal protein L13	1.13	1.62	1.52
	AFLA_134740	60S ribosomal protein L3	1.11	1.51	1.60
	AFLA_079880	60S ribosomal protein L22, putative	1.51	1.95	1.79
	AFLA_029020	40S ribosomal protein S8e	1.06	1.80	1.79
	AFLA_060150	ribosomal protein L26	1.40	1.88	1.87
	AFLA_044530	40S ribosomal protein S15, putative	1.37	1.78	1.53
	AFLA_044520	60S acidic ribosomal protein P2/allergen Asp F 8	2.57	2.34	3.52
	AFLA_025760	50S ribosomal protein L12	1.04	1.46	1.62

	AFLA_086630	60S ribosomal protein L35Ae	1.14	1.86	1.65
	AFLA_127600	40S ribosomal protein S2, putative	1.12	1.45	1.27
	AFLA_112090	60S ribosomal protein L11	1.31	1.64	1.72
	AFLA_041710	60S ribosomal protein L7	1.01	1.68	1.65
	AFLA_103770	60S ribosomal protein L27a, putative	1.15	2.03	1.74
	AFLA_043150	40S ribosomal protein S10a	1.10	2.18	2.10
	AFLA_034340	40S ribosomal protein S10b	1.25	1.98	1.92
	AFLA_125890	40S ribosomal protein S17, putative	1.02	1.74	1.60
	AFLA_033690	60S ribosomal protein L31e	1.41	2.01	2.07
	AFLA_050950	40S ribosomal protein S3, putative	1.04	1.59	1.66
	AFLA_030140	60S ribosomal protein P0	1.14	1.79	1.98
	AFLA_084620	40S ribosomal protein S5, putative	1.09	1.83	1.76
	AFLA_048810	60S ribosomal protein L8, putative	1.31	1.69	1.56
	AFLA_101160	40S ribosomal protein S9	1.00	1.89	1.83
	AFLA_111980	60S ribosomal protein L38, putative	1.38	1.95	1.79
	AFLA_100940	60S ribosomal protein L37	1.43	9.36	4.64
	AFLA_117990	ribosomal protein S5	1.27	1.70	1.79
	AFLA_091250	60S ribosomal protein L24a	1.24	1.69	1.56
	AFLA_075030	40S ribosomal protein S13	1.29	1.58	1.62
	AFLA_044110	40S ribosomal protein Rps16, putative	1.06	1.60	1.50
	AFLA_050650	40S ribosomal protein S19	1.24	1.69	1.56
	AFLA_050000	ribosomal protein L16a	1.02	1.65	1.63
	AFLA_031390	ubiquitin (UbiC), putative	2.39	2.82	1.68
	AFLA_033980	cytosolic large ribosomal subunit protein L7A	1.23	1.69	1.58
	AFLA_127860	60S acidic ribosomal protein P1	1.41	1.58	1.68
RNA transport	AFLA_076980	non-repetitive nucleoporin, putative	1.13	1.58	1.29
	AFLA_069310	conserved hypothetical protein	1.01	1.70	1.41
	AFLA_028910	polyadenylate-binding protein	1.67	1.54	1.32
	AFLA_045330	eukaryotic translation initiation factor subunit eIF-4F, putative	1.55	1.50	1.35
	AFLA_003440	translation initiation factor 4B	1.27	1.78	1.57
	AFLA_081430	eukaryotic translation initiation factor 3 subunit EifCb, putative	1.55	1.50	1.35
	AFLA_041730	eukaryotic translation initiation factor 3 subunit EifCc, putative	1.15	1.21	1.41
	AFLA_086940	eukaryotic translation initiation factor 4, putative	1.66	2.27	2.23
	AFLA_030030	eukaryotic translation initiation factor 3 subunit EifCa, putative	1.57	1.37	1.26

Aminoacyl-tRNA biosynthesis	AFLA_137960	arginyl-tRNA synthetase	1.52	1.57	1.48
mRNA surveillance pathway	AFLA_028910	polyadenylate-binding protein	1.67	1.54	1.32
